# Supplementary material for: The ecomorphology of southern African rodent incisors: Potential applications to the hominin fossil record
Source: PLoS One. 2019 Feb 20;14(2):e0205476. doi: 10.1371/journal.pone.0205476 (PMC6382097; doi:10.1371/journal.pone.0205476)
Supplement: S1 Table — See text for explanation of dietary categories. (DOCX) [file pone.0205476.s001.docx]

| Species | Family | Dietary Category | MD | OD | DE | RC | BD | OA |
| --- | --- | --- | --- | --- | --- | --- | --- | --- |
| *Acomys spinossisimus* | *Muridae* | Seed Eater | 0.655 | 1.260 | 0.378 | 2.955 | 1.227 | 20 |
| *Acomys spinossisimus* | *Muridae* | Seed Eater | 0.635 | 1.369 | 0.417 | 2.910 | 1.276 | 21 |
| *Acomys spinossisimus* | *Muridae* | Seed Eater | 0.735 | 1.371 | 0.261 | 3.105 | 1.254 | 22 |
| *Acomys spinossisimus* | *Muridae* | Seed Eater | 0.670 | 1.492 | 0.371 | 2.970 | 1.359 | 19 |
| *Acomys subspinosus* | *Muridae* | Seed Eater | 0.540 | 1.594 | 0.467 | 3.095 | 1.572 | 12 |
| *Acomys subspinosus* | *Muridae* | Seed Eater | 0.560 | 1.372 | 0.350 | 2.794 | 1.262 | 22 |
| *Acomys subspinosus* | *Muridae* | Seed Eater | 0.665 | 1.543 | 0.470 | 3.267 | 1.416 | 26 |
| *Acomys subspinosus* | *Muridae* | Seed Eater | 0.660 | 1.766 | 0.456 | 3.054 | 1.433 | 26 |
| *Aethomys chrysophilus* | *Muridae* | Omnivore | 1.070 | 2.180 | 0.655 | 4.945 | 1.964 | 25 |
| *Aethomys chrysophilus* | *Muridae* | Omnivore | 1.180 | 2.237 | 0.815 | 5.005 | 2.083 | 22 |
| *Aethomys chrysophilus* | *Muridae* | Omnivore | 1.200 | 2.232 | 0.570 | 5.288 | 2.115 | 18 |
| *Aethomys chrysophilus* | *Muridae* | Omnivore | 1.205 | 2.322 | 0.726 | 5.053 | 2.039 | 26 |
| *Cricetomys gambianus* | *Nesomyidae* | Herbivore | 2.880 | 6.133 | 1.068 | 11.258 | 3.900 | 42 |
| *Cricetomys gambianus* | *Nesomyidae* | Herbivore | 3.070 | 5.703 | 1.202 | 12.269 | 4.187 | 43 |
| *Cricetomys gambianus* | *Nesomyidae* | Herbivore | 2.800 | 5.348 | 1.108 | 12.039 | 3.867 | 47 |
| *Cricetomys gambianus* | *Nesomyidae* | Herbivore | 2.610 | 5.087 | 1.095 | 10.980 | 3.658 | 38 |
| *Cryptomys hottentotus* | *Bathyergidae* | Herbivore | 2.010 | 4.993 | 1.103 | 11.182 | 1.928 | 56 |
| *Cryptomys hottentotus* | *Bathyergidae* | Herbivore | 2.250 | 4.815 | 1.200 | 11.671 | 2.363 | 53 |
| *Cryptomys hottentotus* | *Bathyergidae* | Herbivore | 2.130 | 5.097 | 1.161 | 12.741 | 2.101 | 57 |
| *Cryptomys hottentotus* | *Bathyergidae* | Herbivore | 2.180 | 4.785 | 1.074 | 11.575 | 2.142 | 57 |
| *Dasymys incomptus* | *Muridae* | Herbivore | 1.730 | 2.913 | 1.052 | 5.945 | 2.314 | 30 |
| *Dasymys incomptus* | *Muridae* | Herbivore | 1.810 | 3.528 | 0.977 | 6.834 | 2.459 | 37 |
| *Dasymys incomptus* | *Muridae* | Herbivore | 1.770 | 3.823 | 1.094 | 6.618 | 2.444 | 36 |
| *Dasymys incomptus* | *Muridae* | Herbivore | 1.780 | 3.166 | 1.016 | 6.219 | 2.322 | 33 |
| *Dendromus melanotis* | *Nesomyidae* | Seed Eater | 0.660 | 1.392 | 0.563 | 2.694 | 1.151 | 27 |
| *Dendromus melanotis* | *Nesomyidae* | Seed Eater | 0.690 | 1.246 | 0.476 | 2.776 | 1.117 | 24 |
| *Dendromus melanotis* | *Nesomyidae* | Seed Eater | 0.675 | 1.430 | 0.547 | 2.729 | 1.100 | 31 |
| *Dendromus melanotis* | *Nesomyidae* | Seed Eater | 0.685 | 1.300 | 0.561 | 2.557 | 1.116 | 26 |
| *Dendromus mesomelas* | *Nesomyidae* | Seed Eater | 0.625 | 1.508 | 0.618 | 2.872 | 1.277 | 29 |
| *Dendromus mesomelas* | *Nesomyidae* | Seed Eater | 0.690 | 1.486 | 0.517 | 3.035 | 1.187 | 32 |
| *Dendromus mesomelas* | *Nesomyidae* | Seed Eater | 0.720 | 1.644 | 0.585 | 3.128 | 1.242 | 34 |
| *Dendromus mesomelas* | *Nesomyidae* | Seed Eater | 0.715 | 1.467 | 0.583 | 2.958 | 1.213 | 29 |
| *Dendromus mysticalis* | *Nesomyidae* | Seed Eater | 0.585 | 1.225 | 0.589 | 2.814 | 1.129 | 22 |
| *Dendromus mysticalis* | *Nesomyidae* | Seed Eater | 0.515 | 1.110 | 0.510 | 2.411 | 0.988 | 25 |
| *Dendromus mysticalis* | *Nesomyidae* | Seed Eater | 0.730 | 1.524 | 0.594 | 2.715 | 1.145 | 31 |
| *Dendromus mysticalis* | *Nesomyidae* | Seed Eater | 0.630 | 1.376 | 0.629 | 2.576 | 1.153 | 25 |
| *Desmodillus auricularis* | *Muridae* | Seed Eater | 0.810 | 2.452 | 0.551 | 5.041 | 1.927 | 32 |
| *Desmodillus auricularis* | *Muridae* | Seed Eater | 0.930 | 2.823 | 0.630 | 5.599 | 2.299 | 28 |
| *Desmodillus auricularis* | *Muridae* | Seed Eater | 1.000 | 3.061 | 0.503 | 5.759 | 2.216 | 33 |
| *Desmodillus auricularis* | *Muridae* | Seed Eater | 0.720 | 2.166 | 0.511 | 4.652 | 1.669 | 33 |
| *Georychus capensis* | *Bathyergidae* | Herbivore | 2.690 | 5.790 | 1.347 | 16.085 | 2.681 | 58 |
| *Georychus capensis* | *Bathyergidae* | Herbivore | 2.945 | 5.983 | 1.867 | 19.835 | 3.140 | 61 |
| *Georychus capensis* | *Bathyergidae* | Herbivore | 2.340 | 5.269 | 1.200 | 17.403 | 2.520 | 58 |
| *Gerbillurus paeba* | *Muridae* | Seed Eater | 0.755 | 2.287 | 0.495 | 4.412 | 1.471 | 41 |
| *Gerbillurus paeba* | *Muridae* | Seed Eater | 0.800 | 2.020 | 0.599 | 3.886 | 1.526 | 33 |
| *Gerbillurus paeba* | *Muridae* | Seed Eater | 0.845 | 2.001 | 0.583 | 4.219 | 1.581 | 33 |
| *Gerbillurus paeba* | *Muridae* | Seed Eater | 0.655 | 1.806 | 0.444 | 3.376 | 1.447 | 34 |
| *Grammomys dolichurus* | *Muridae* | Omnivore | 0.850 | 1.915 | 0.423 | 3.465 | 1.506 | 28 |
| *Grammomys dolichurus* | *Muridae* | Omnivore | 1.125 | 2.466 | 0.587 | 4.735 | 1.872 | 33 |
| *Grammomys dolichurus* | *Muridae* | Omnivore | 0.970 | 2.430 | 0.428 | 4.727 | 1.857 | 31 |
| *Grammomys dolichurus* | *Muridae* | Omnivore | 1.125 | 2.614 | 0.396 | 5.151 | 2.089 | 30 |
| *Graphiuris murinus* | *Myoxidae* | Omnivore | 0.840 | 1.556 | 0.267 | 2.829 | 1.029 | 38 |
| *Graphiuris murinus* | *Myoxidae* | Omnivore | 1.030 | 2.261 | 0.305 | 4.137 | 1.498 | 37 |
| *Graphiuris murinus* | *Myoxidae* | Omnivore | 0.960 | 2.338 | 0.277 | 4.168 | 1.442 | 40 |
| *Graphiuris murinus* | *Myoxidae* | Omnivore | 1.025 | 2.141 | 0.294 | 4.247 | 1.477 | 37 |
| *Lemniscomys rosalia* | *Muridae* | Omnivore | 1.400 | 2.294 | 0.591 | 5.017 | 1.852 | 29 |
| *Lemniscomys rosalia* | *Muridae* | Omnivore | 1.185 | 2.235 | 0.674 | 4.520 | 1.770 | 31 |
| *Lemniscomys rosalia* | *Muridae* | Omnivore | 1.340 | 2.688 | 0.588 | 5.182 | 1.883 | 34 |
| *Lemniscomys rosalia* | *Muridae* | Omnivore | 0.735 | 2.079 | 0.483 | 3.968 | 1.681 | 29 |
| *Malacothrix typica* | *Nesomyidae* | Omnivore | 0.645 | 1.618 | 0.738 | 3.803 | 1.279 | 32 |
| *Malacothrix typica* | *Nesomyidae* | Omnivore | 0.650 | 1.423 | 0.652 | 3.299 | 1.121 | 32 |
| *Malacothrix typica* | *Nesomyidae* | Omnivore | 0.685 | 1.691 | 0.692 | 3.599 | 1.302 | 31 |
| *Malacothrix typica* | *Nesomyidae* | Omnivore | 0.620 | 1.487 | 0.667 | 3.607 | 1.239 | 33 |
| *Mastomys coucha* | *Muridae* | Omnivore | 0.925 | 2.144 | 0.400 | 4.591 | 1.885 | 24 |
| *Mastomys coucha* | *Muridae* | Omnivore | 0.925 | 2.211 | 0.417 | 4.202 | 1.840 | 28 |
| *Mastomys coucha* | *Muridae* | Omnivore | 0.760 | 1.924 | 0.339 | 4.017 | 1.660 | 29 |
| *Mastomys coucha* | *Muridae* | Omnivore | 0.760 | 1.941 | 0.351 | 3.664 | 1.564 | 31 |
| *Mastomys natalensis* | *Muridae* | Omnivore | 0.750 | 1.658 | 0.407 | 3.408 | 1.540 | 18 |
| *Mastomys natalensis* | *Muridae* | Omnivore | 0.805 | 1.957 | 0.416 | 3.643 | 1.544 | 30 |
| *Mastomys natalensis* | *Muridae* | Omnivore | 0.520 | 1.610 | 0.303 | 3.036 | 1.344 | 30 |
| *Mastomys natalensis* | *Muridae* | Omnivore | 0.540 | 1.492 | 0.398 | 2.987 | 1.251 | 27 |
| *Micaelamys namaquensis* | *Muridae* | Omnivore | 1.185 | 2.245 | 0.608 | 5.213 | 2.184 | 18 |
| *Micaelamys namaquensis* | *Muridae* | Omnivore | 0.860 | 1.703 | 0.547 | 3.375 | 1.459 | 30 |
| *Micaelamys namaquensis* | *Muridae* | Omnivore | 1.095 | 2.155 | 0.599 | 4.570 | 1.721 | 29 |
| *Micaelamys namaquensis* | *Muridae* | Omnivore | 1.110 | 2.332 | 0.577 | 4.623 | 1.857 | 29 |
| *Mus indutus* | *Muridae* | Seed Eater | 0.480 | 1.187 | 0.256 | 2.426 | 0.922 | 32 |
| *Mus indutus* | *Muridae* | Seed Eater | 0.460 | 0.966 | 0.217 | 2.081 | 0.869 | 24 |
| *Mus indutus* | *Muridae* | Seed Eater | 0.540 | 1.234 | 0.246 | 2.429 | 1.044 | 26 |
| *Mus indutus* | *Muridae* | Seed Eater | 0.500 | 1.013 | 0.209 | 2.336 | 0.924 | 23 |
| *Mus minutoides* | *Muridae* | Omnivore | 0.465 | 0.837 | 0.229 | 1.956 | 0.784 | 28 |
| *Mus minutoides* | *Muridae* | Omnivore | 0.490 | 0.954 | 0.213 | 2.219 | 0.893 | 22 |
| *Mus minutoides* | *Muridae* | Omnivore | 0.440 | 0.941 | 0.251 | 2.164 | 0.948 | 8 |
| *Mus minutoides* | *Muridae* | Omnivore | 0.470 | 0.930 | 0.222 | 2.136 | 0.889 | 18 |
| *Mystromys albicaudatus* | *Nesomyidae* | Omnivore | 1.220 | 3.037 | 0.758 | 5.240 | 2.089 | 36 |
| *Mystromys albicaudatus* | *Nesomyidae* | Omnivore | 1.290 | 2.921 | 0.598 | 4.973 | 1.944 | 35 |
| *Mystromys albicaudatus* | *Nesomyidae* | Omnivore | 1.510 | 3.045 | 0.670 | 5.806 | 2.083 | 35 |
| *Mystromys albicaudatus* | *Nesomyidae* | Omnivore | 1.430 | 2.754 | 0.603 | 5.901 | 1.989 | 36 |
| *Otomys angoniensis* | *Muridae* | Herbivore | 2.060 | 2.405 | 0.672 | 5.817 | 2.107 | 27 |
| *Otomys angoniensis* | *Muridae* | Herbivore | 1.850 | 2.327 | 0.629 | 6.044 | 1.894 | 32 |
| *Otomys angoniensis* | *Muridae* | Herbivore | 1.895 | 2.386 | 0.710 | 5.801 | 2.063 | 27 |
| *Otomys angoniensis* | *Muridae* | Herbivore | 2.005 | 2.443 | 0.652 | 5.881 | 2.102 | 30 |
| *Otomys irroratus* | *Muridae* | Herbivore | 1.770 | 2.493 | 0.723 | 5.534 | 1.884 | 36 |
| *Otomys irroratus* | *Muridae* | Herbivore | 2.205 | 3.031 | 0.962 | 6.705 | 2.332 | 37 |
| *Otomys irroratus* | *Muridae* | Herbivore | 1.985 | 2.342 | 0.601 | 6.078 | 1.919 | 34 |
| *Otomys irroratus* | *Muridae* | Herbivore | 1.450 | 1.675 | 0.700 | 4.780 | 1.408 | 34 |
| *Otomys sloggetti* | *Muridae* | Herbivore | 2.218 | 4.887 | 0.945 | 8.302 | 2.636 | 53 |
| *Otomys sloggetti* | *Muridae* | Herbivore | 2.125 | 4.415 | 1.009 | 7.733 | 2.725 | 42 |
| *Otomys sloggetti* | *Muridae* | Herbivore | 1.420 | 2.479 | 0.706 | 4.864 | 1.654 | 40 |
| *Otomys sloggetti* | *Muridae* | Herbivore | 2.000 | 3.172 | 0.973 | 7.360 | 2.371 | 42 |
| *Paraxerus cepapi* | *Sciuridae* | Omnivore | 1.185 | 2.983 | 0.545 | 6.991 | 2.604 | 27 |
| *Paraxerus cepapi* | *Sciuridae* | Omnivore | 1.385 | 3.097 | 0.605 | 7.248 | 2.656 | 30 |
| *Paraxerus cepapi* | *Sciuridae* | Omnivore | 1.180 | 3.320 | 0.640 | 6.349 | 2.612 | 29 |
| *Paraxerus cepapi* | *Sciuridae* | Omnivore | 1.425 | 2.784 | 0.662 | 7.389 | 2.711 | 27 |
| *Paraxerus palliatus* | *Sciuridae* | Omnivore | 1.345 | 3.484 | 0.650 | 8.237 | 2.681 | 34 |
| *Paraxerus palliatus* | *Sciuridae* | Omnivore | 1.335 | 3.203 | 0.552 | 7.207 | 2.606 | 32 |
| *Paraxerus palliatus* | *Sciuridae* | Omnivore | 1.305 | 3.498 | 0.442 | 7.764 | 2.863 | 27 |
| *Paraxerus palliatus* | *Sciuridae* | Omnivore | 1.410 | 2.465 | 0.452 | 7.251 | 2.962 | 32 |
| *Parotomys littledalei* | *Muridae* | Herbivore | 1.440 | 3.102 | 0.415 | 6.044 | 2.103 | 38 |
| *Parotomys littledalei* | *Muridae* | Herbivore | 1.300 | 3.291 | 0.445 | 6.180 | 1.869 | 43 |
| *Parotomys littledalei* | *Muridae* | Herbivore | 1.085 | 2.807 | 0.391 | 5.395 | 1.784 | 40 |
| *Parotomys littledalei* | *Muridae* | Herbivore | 1.150 | 2.645 | 0.352 | 5.290 | 1.714 | 39 |
| *Pedetes capensis* | *Pedetidae* | Herbivore | 4.315 | 7.123 | 1.822 | 19.148 | 4.570 | 42 |
| *Pedetes capensis* | *Pedetidae* | Herbivore | 3.510 | 4.695 | 1.396 | 15.526 | 3.861 | 38 |
| *Pedetes capensis* | *Pedetidae* | Herbivore | 4.010 | 6.217 | 1.931 | 17.788 | 4.510 | 39 |
| *Pedetes capensis* | *Pedetidae* | Herbivore | 3.820 | 5.879 | 1.827 | 16.708 | 4.521 | 37 |
| *Pelomys fallax* | *Muridae* | Herbivore | 1.670 | 3.369 | 0.722 | 6.196 | 2.146 | 38 |
| *Pelomys fallax* | *Muridae* | Herbivore | 1.235 | 2.193 | 0.701 | 4.389 | 1.742 | 32 |
| *Pelomys fallax* | *Muridae* | Herbivore | 1.150 | 2.308 | 0.796 | 4.220 | 1.750 | 32 |
| *Pelomys fallax* | *Muridae* | Herbivore | 1.625 | 2.884 | 0.678 | 5.330 | 1.885 | 37 |
| *Petromyscus collinus* | *Nesomyidae* | Seed Eater | 0.550 | 1.398 | 0.335 | 3.109 | 1.293 | 23 |
| *Petromyscus collinus* | *Nesomyidae* | Seed Eater | 0.665 | 1.673 | 0.390 | 3.095 | 1.303 | 29 |
| *Petromyscus collinus* | *Nesomyidae* | Seed Eater | 0.540 | 1.357 | 0.335 | 2.943 | 1.242 | 23 |
| *Petromyscus collinus* | *Nesomyidae* | Seed Eater | 0.650 | 1.594 | 0.398 | 3.067 | 1.454 | 19 |
| *Rhabdomys pumilio* | *Muridae* | Omnivore | 1.100 | 2.194 | 0.586 | 4.025 | 1.678 | 29 |
| *Rhabdomys pumilio* | *Muridae* | Omnivore | 0.895 | 1.808 | 0.332 | 3.968 | 1.538 | 29 |
| *Rhabdomys pumilio* | *Muridae* | Omnivore | 0.895 | 1.850 | 0.386 | 3.650 | 1.403 | 33 |
| *Rhabdomys pumilio* | *Muridae* | Omnivore | 0.960 | 2.392 | 0.454 | 3.640 | 1.537 | 34 |
| *Saccostomys campestris* | *Nesomyidae* | Seed Eater | 0.900 | 2.008 | 0.389 | 3.952 | 1.305 | 38 |
| *Saccostomys campestris* | *Nesomyidae* | Seed Eater | 0.920 | 1.972 | 1.026 | 3.987 | 1.485 | 36 |
| *Saccostomys campestris* | *Nesomyidae* | Seed Eater | 1.080 | 2.453 | 0.549 | 4.501 | 1.876 | 33 |
| *Saccostomys campestris* | *Nesomyidae* | Seed Eater | 1.200 | 2.837 | 0.585 | 4.835 | 1.761 | 38 |
| *Steatomys krebsii* | *Nesomyidae* | Seed Eater | 0.845 | 1.452 | 0.698 | 3.879 | 1.320 | 31 |
| *Steatomys krebsii* | *Nesomyidae* | Seed Eater | 0.650 | 1.193 | 0.562 | 2.803 | 1.099 | 23 |
| *Steatomys krebsii* | *Nesomyidae* | Seed Eater | 0.770 | 1.469 | 0.643 | 3.367 | 1.213 | 29 |
| *Steatomys krebsii* | *Nesomyidae* | Seed Eater | 0.780 | 1.589 | 0.641 | 3.638 | 1.211 | 36 |
| *Steatomys parvus* | *Nesomyidae* | Seed Eater | 0.710 | 1.496 | 0.747 | 3.629 | 1.264 | 28 |
| *Steatomys parvus* | *Nesomyidae* | Seed Eater | 0.795 | 1.577 | 0.613 | 3.140 | 1.235 | 30 |
| *Steatomys parvus* | *Nesomyidae* | Seed Eater | 0.710 | 1.697 | 0.718 | 3.312 | 1.283 | 33 |
| *Steatomys parvus* | *Nesomyidae* | Seed Eater | 0.726 | 1.695 | 0.868 | 3.349 | 1.269 | 32 |
| *Steatomys pratensis* | *Nesomyidae* | Seed Eater | 0.785 | 1.484 | 0.559 | 3.421 | 1.156 | 33 |
| *Steatomys pratensis* | *Nesomyidae* | Seed Eater | 0.710 | 1.637 | 0.613 | 3.917 | 1.214 | 39 |
| *Steatomys pratensis* | *Nesomyidae* | Seed Eater | 0.695 | 1.354 | 0.688 | 3.154 | 1.148 | 27 |
| *Steatomys pratensis* | *Nesomyidae* | Seed Eater | 0.795 | 1.112 | 0.590 | 3.379 | 1.133 | 25 |
| *Tatera brantsii* | *Muridae* | Omnivore | 1.255 | 2.782 | 0.962 | 5.537 | 2.199 | 31 |
| *Tatera brantsii* | *Muridae* | Omnivore | 1.300 | 2.463 | 0.753 | 5.952 | 2.129 | 28 |
| *Tatera brantsii* | *Muridae* | Omnivore | 1.330 | 2.481 | 1.075 | 5.994 | 2.099 | 23 |
| *Tatera brantsii* | *Muridae* | Omnivore | 1.300 | 2.923 | 0.975 | 6.157 | 2.363 | 31 |
| *Tatera leucogaster* | *Muridae* | Omnivore | 1.220 | 2.618 | 0.852 | 5.507 | 2.176 | 28 |
| *Tatera leucogaster* | *Muridae* | Omnivore | 1.305 | 2.693 | 0.872 | 5.677 | 2.114 | 31 |
| *Tatera leucogaster* | *Muridae* | Omnivore | 1.155 | 2.260 | 0.848 | 4.870 | 2.132 | 26 |
| *Tatera leucogaster* | *Muridae* | Omnivore | 1.145 | 2.622 | 0.920 | 5.750 | 2.123 | 31 |
| *Thallomys paedulcus* | *Muridae* | Seed Eater | 0.830 | 1.727 | 0.446 | 4.513 | 1.561 | 25 |
| *Thallomys paedulcus* | *Muridae* | Seed Eater | 0.905 | 2.346 | 0.544 | 4.935 | 1.808 | 32 |
| *Thallomys paedulcus* | *Muridae* | Seed Eater | 0.850 | 1.996 | 0.558 | 4.811 | 1.840 | 27 |
| *Thallomys paedulcus* | *Muridae* | Seed Eater | 0.810 | 1.984 | 0.522 | 4.322 | 1.691 | 29 |
| *Xerus inauris* | *Sciuridae* | Herbivore | 1.850 | 4.843 | 0.987 | 10.282 | 3.739 | 32 |
| *Xerus inauris* | *Sciuridae* | Herbivore | 1.860 | 3.941 | 0.888 | 9.924 | 3.642 | 21 |
| *Xerus inauris* | *Sciuridae* | Herbivore | 1.650 | 4.510 | 0.941 | 9.187 | 3.349 | 34 |
| *Xerus inauris* | *Sciuridae* | Herbivore | 1.850 | 4.231 | 0.899 | 10.228 | 3.353 | 31 |
| *Zelotomys woosnami* | *Muridae* | Omnivore | 1.150 | 3.195 | 0.544 | 5.906 | 2.142 | 37 |
| *Zelotomys woosnami* | *Muridae* | Omnivore | 1.450 | 2.902 | 0.693 | 5.615 | 2.453 | 25 |
| *Zelotomys woosnami* | *Muridae* | Omnivore | 1.100 | 2.973 | 0.677 | 4.896 | 2.063 | 33 |
| *Zelotomys woosnami* | *Muridae* | Omnivore | 1.330 | 2.851 | 0.742 | 6.094 | 2.231 | 35 |
