# Supplementary material for: The ecomorphology of southern African rodent incisors: Potential applications to the hominin fossil record
Source: PLoS One. 2019 Feb 20;14(2):e0205476. doi: 10.1371/journal.pone.0205476 (PMC6382097; doi:10.1371/journal.pone.0205476)
Supplement: S2 Table — Values > 1 suggest that phylogeny is driving the similarities between closely related species. Only values < 1 were used in our analysis. (DOCX) [file pone.0205476.s002.docx]

| **Measurement** | **Blomberg's *K*** |
| --- | --- |
| OD/MD | 0.495 |
| MD/OD | 0.516 |
| OA/DE | 0.686 |
| OA/BD | 0.825 |
| OA/MD | 0.846 |
| OD/DE | 0.864 |
| OD/RC | 0.864 |
| MD/RC | 0.867 |
| BD/MD | 0.871 |
| BD/DE | 0.884 |
| RC/MD | 0.915 |
| MD/DE | 0.916 |
| DE/MD | 0.962 |
| RC/OD | 0.965 |
| DE/OA | 1.081 |
| DE/BD | 1.106 |
| DE/OD | 1.118 |
| MD/OA | 1.154 |
| OA/OD | 1.157 |
| BD/OD | 1.220 |
| RC/DE | 1.222 |
| DE/RC | 1.235 |
| MD/BD | 1.246 |
| OA/RC | 1.255 |
| BD/OA | 1.257 |
| DE | 1.420 |
| OD/OA | 1.508 |
| BD | 1.521 |
| OA | 1.680 |
| MD | 1.715 |
| OD/BD | 1.908 |
| OD | 1.910 |
| RC/OA | 1.970 |
| RC | 2.464 |
| BD/RC | 2.603 |
| RC/BD | 3.433 |
