# Supplementary material for: The ecomorphology of southern African rodent incisors: Potential applications to the hominin fossil record
Source: PLoS One. 2019 Feb 20;14(2):e0205476. doi: 10.1371/journal.pone.0205476 (PMC6382097; doi:10.1371/journal.pone.0205476)
Supplement: S3 Table — Misclassifications are in bold. See text for explanation of dietary categories. (DOCX) [file pone.0205476.s003.docx]

| **Species** | **Actual Diet** | **Predicted** |
| --- | --- | --- |
| *Micaelamys namaquensis* | Omnivore | Omnivore |
| *Micaelamys namaquensis* | **Omnivore** | **Herbivore** |
| *Micaelamys namaquensis* | Omnivore | Omnivore |
| *Micaelamys namaquensis* | **Omnivore** | **Herbivore** |
| *Tatera leucogaster* | Omnivore | Omnivore |
| *Tatera leucogaster* | Omnivore | Omnivore |
| *Tatera leucogaster* | Omnivore | Omnivore |
| *Tatera leucogaster* | Omnivore | Omnivore |
| *Otomys irroratus* | Herbivore | Herbivore |
| *Otomys irroratus* | Herbivore | Herbivore |
| *Otomys irroratus* | Herbivore | Herbivore |
| *Otomys irroratus* | Herbivore | Herbivore |
| *Rhabdomys pumilio* | **Omnivore** | **Herbivore** |
| *Rhabdomys pumilio* | Omnivore | Omnivore |
| *Rhabdomys pumilio* | Omnivore | Omnivore |
| *Rhabdomys pumilio* | Omnivore | Omnivore |
| *Mastomys coucha* | **Omnivore** | **Seed Eater** |
| *Mastomys coucha* | Omnivore | Omnivore |
| *Mastomys coucha* | Omnivore | Omnivore |
| *Mastomys coucha* | Omnivore | Omnivore |
| *Dendromus mysticalis* | Seed Eater | Seed Eater |
| *Dendromus mysticalis* | Seed Eater | Seed Eater |
| *Dendromus mysticalis* | Seed Eater | Seed Eater |
| *Dendromus mysticalis* | Seed Eater | Seed Eater |
